# Kansas Veterinary Medical Association

**Published:** 1891-11

**Authors:** N. S. Mayo

**Affiliations:** Secretary


*Kansas Veterinary Medical Association.*—The annual meeting of the Kansas Veterinary Medical Association was held at Topeka, Kansas, September 17, 1891, at "The Copeland." The meeting was called to order by Pres. Pritchard. Minutes of previous meeting read and approved. Officers for the ensuing year were elected as follows: President, Dr. George C.

Pritchard, of Topeka; Vice-President, Dr. S. L. Hunter, of Fort Leavenworth; Secretary, Dr. N. S. Mayo, of Manhattan; Treasurer, Dr. W. H. Richards, of Emporia. Board of Censors: Dr. J. M. Phillips, of Wichita. Dr. D. Le May, of Fort Riley, and Dr. L. R. Brady, of Manhattan.

The society then passed to the head of unfinished business. The resignation of Dr. C. S. Orr was laid upon the table until the next meeting. Drs. Hunter, Richards and Brady gave notice that a request would be made at the next meeting for a change of Sec. IV, of the Code of Ethics.

A paper on "Bursattee" was presented by Dr. S. E. Phillips, of Wichita, which was very interesting and thoroughly discussed. The subjects of Bovine Tuberculosis, Enzootic Cerebretis, and Hernia were brought up, discussed, and cases reported. Drs. Orr, Brady and Mayo extended an invitation for the association to hold its next meeting at Manhattan. The invitation was accepted and it was decided to make the meeting there the "banner" meeting, both in attendance and interest. The society then adjourned to meet in Manhattan the second Thursday in March, 1892.

N. S. Mayo, *Secretary.*

